# Sophoridine Inhibits the Tumour Growth of Non-Small Lung Cancer by Inducing Macrophages M1 Polarisation *via* MAPK-Mediated Inflammatory Pathway

**DOI:** 10.3389/fonc.2021.634851

**Published:** 2021-02-24

**Authors:** Bei Zhao, Xiaodan Hui, Hairong Zeng, Yinan Yin, Jian Huang, Qingfeng Tang, Guangbo Ge, Tao Lei

**Affiliations:** ^1^ Putuo Hospital, Shanghai University of Traditional Chinese Medicine, Shanghai, China; ^2^ Institute of Interdisciplinary Integrative Medicine Research, Shanghai University of Traditional Chinese Medicine, Shanghai, China; ^3^ Department of Wine, Food, and Molecular Bioscience, Faculty of Life Science, Lincoln University, Christchurch, New Zealand; ^4^ Pharmacology and Toxicology Division, Shanghai Institute of Food and Drug Control, Shanghai, China

**Keywords:** macrophage differentiation, mitogen-activated protein kinase (MPKs) pathway, sophoridine, RAW264.7, THP-1 cells

## Abstract

Lung cancer is one of the most common and lethal neoplasms for which very few efficacious treatments are currently available. M1-like polarised tumour-associated macrophages (TAMs) are key mediators to modulate the tumour microenvironment, which play a key role in inhibiting cancer cell growth. Sophoridine, a naturally occurring alkaloid, exerts multiple pharmacological activities including anti-tumour and anti-inflammatory activities, but it has not been characterised as a regulator of tumour microenvironment towards NSCLC. Herein, the regulatory effects of sophoridine on the polarisation of THP-1 cells into TAMs and the anti-tumour effects of sophoridine-stimulated M1 polarised macrophages towards lung cancer cells were carefully investigated both *in vitro* and *in vivo*. The results showed that sophoridine could significantly promote M1 polarisation of RAW264.7 and THP-1-derived macrophages, leading to increased expression of pro-inflammatory cytokines and the M1 surface markers CD86 *via* activating MAPKs signaling pathway. Further investigations showed that sophoridine-stimulated RAW264.7 and THP-1-derived M1 macrophages effectively induced cell apoptosis as well as inhibited the cell colony formation and cell proliferation in both H460 and Lewis lung cancer cells. In Lewis-bearing mice model, sophoridine (15 or 25 mg/kg) significantly inhibited the tumour growth and up-regulated the expression of CD86/F4/80 in tumour tissues. Collectively, the findings clearly demonstrate that sophoridine promoted M1-like polarisation *in vitro* and *in vivo*, suggesting that sophoridine held a great therapeutic potential for treating lung cancer.

## Introduction

Lung cancer is one of the leading causes of cancer-related mortality over the world, and its 5-year survival rate is less than 18% (1). Approximately 1.8 million lung cancer patients are diagnosed annually, 80% of which present with an advanced stage disease (2). 80% of lung cancer cases are non-small cell lung cancer (NSCLC). The prognosis of NSCLC patients remains poor in spite of several advances in early detection and systematic therapies. The high mortality rate of NSCLC is mainly responsible for the difficulty of early detection for prognosis, high risk of metastasis, and inadequate reactions to chemical therapy and radiotherapy (3). Since no curative therapy has been developed to date for the developed lung cancer, clinical care is mostly palliative (4). It is therefore necessary to gain deep insight into the fundamental biological and molecular mechanisms of the progression of NSCLC.

Intercommunication between the tumour and its microenvironment contributes to several steps of cancer development (5). Tumour-associated macrophages (TAMs) are the macrophages that migrate to the stroma of tumour (6). Both tissue-resident macrophages and monocyte-derived macrophages are employed during the inflammation (7). Classically activated macrophages (M1s) and the alternatively active macrophages (M2s) are two major macrophage polarisation states and also represent the Th1/Th2 differentiation paradigm (8). Th1-related cytokines, including LPS, polarise macrophages to the M1 phenotype, producing a high level of pro-inflammatory cytokines, such as IL-6, IL-1β, TNF-α, reactive oxygen, and nitrogen species (9, 10). In comparison, IL-4 activates macrophages to differentiate into M2 phenotype, exerting anti-inflammatory and pro-tumourigenic properties (11, 12). MAPKs signalling pathway is involved in the inflammatory activation of macrophages and reprogramming TAMs towards the M1 phenotype (13, 14). Depletion of TAMs or inducing macrophage M1 polarisation status *via* MAPKs signalling pathway is taken to be a promising therapeutic strategy for treating NSCLC.

**Figure 1:**
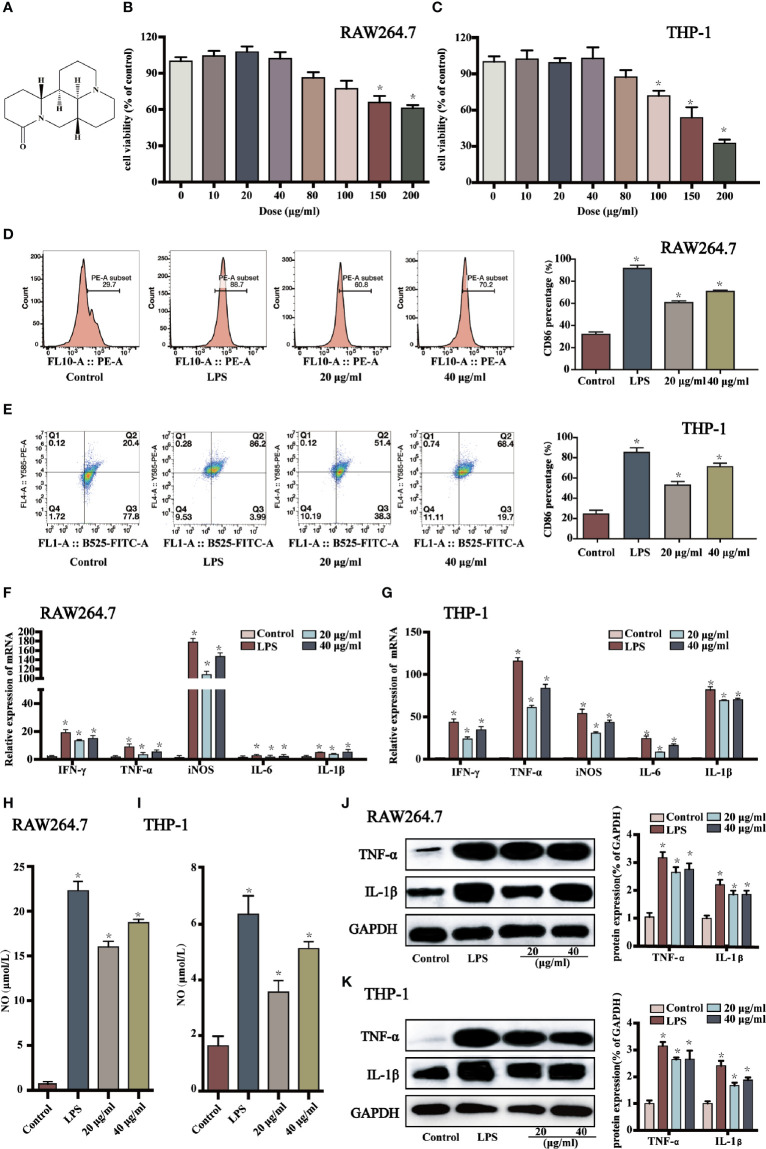
Sophoridine promoted macrophages shifting to M1 phenotype. **(A)** Chemical structure of sophoridine (downloaded from PubChem). **(B, C)** The cell viability of sophoridine-stimulated RAW264.7 and THP-1-derived macrophages was detected by CCK-8 assay. **(D, E)** The percentage of CD86 of RAW264.7 and THP-1-derived macrophages. **(F, G)** The relative expression of IFN-*γ*, TNF-α, IL-6, iNOS, and IL-1β mRNA in RAW264.7 and THP-1-derived macrophages determined by RT-PCR. **(H, I)** The NO production of RAW264.7 and THP-1-derived macrophages assessed by Griess. **(J, K)** The protein expression (% of GAPDH) of TNF-α and IL-1β in RAW264.7 and THP-1-derived macrophages determine by Western blotting (n = 3), *p* < 0.05 (*).

The combination of traditional therapies with natural compounds has shown an additive effect due to the alternative activation of the signalling pathway, which can lead to cell death or improve the efficiency of the chemotherapeutic agents (15). The participation in cancer immunobiology of these natural compounds (either alone or in combination therapy) may offer potential therapeutic possibilities (16). Herbal medicines contain numerous of biologically active natural compounds, which have been reported to have remarkable therapeutic efficacy with minimal adverse effects and also as a major source for discovery of drug lead compounds (17). Sophoridine (C_15_H_24_N_2_O, **Figure 1A**) is a bioactive quinolizidine alkaloid isolated from the leaves of Sophora *alopecuroides. L* (18). Studies have revealed that sophoridine exhibited impressive viral pharmacological effects, including anti-inflammatory, anti-virus, and anti-cancer effects (19). The anti-tumour effects of sophoridine are involved in several underlying mechanisms, including arresting the cell cycle of pancreatic cancer cells in the S or G0/G1 phase through activation of the phosphorylation of MAPK signalling pathways (20), inhibiting the tumour progression and invasion in human colorectal cancer cells (21), and inhibiting ubiquitin-proteasome signalling pathway in human glioma cells (22). Sophoridine has been reported (23) to have the ability of forming the gastric cancer immune microenvironment by transferring TAM polarisation to M1 and suppressing M2-TAM polarisation. However, only one study (24) reported that sophoridine exerted anti-tumour effects *via* activation of p53 and Hippo signalling pathways towards lung cancer cells. It has not been reported whether sophoridine could inhibit the growth of lung cancer cells *via* promoting TAM polarisation to pro-inflammatory M1 status or regulating the tumour microenvironment.

**Figure 2:**
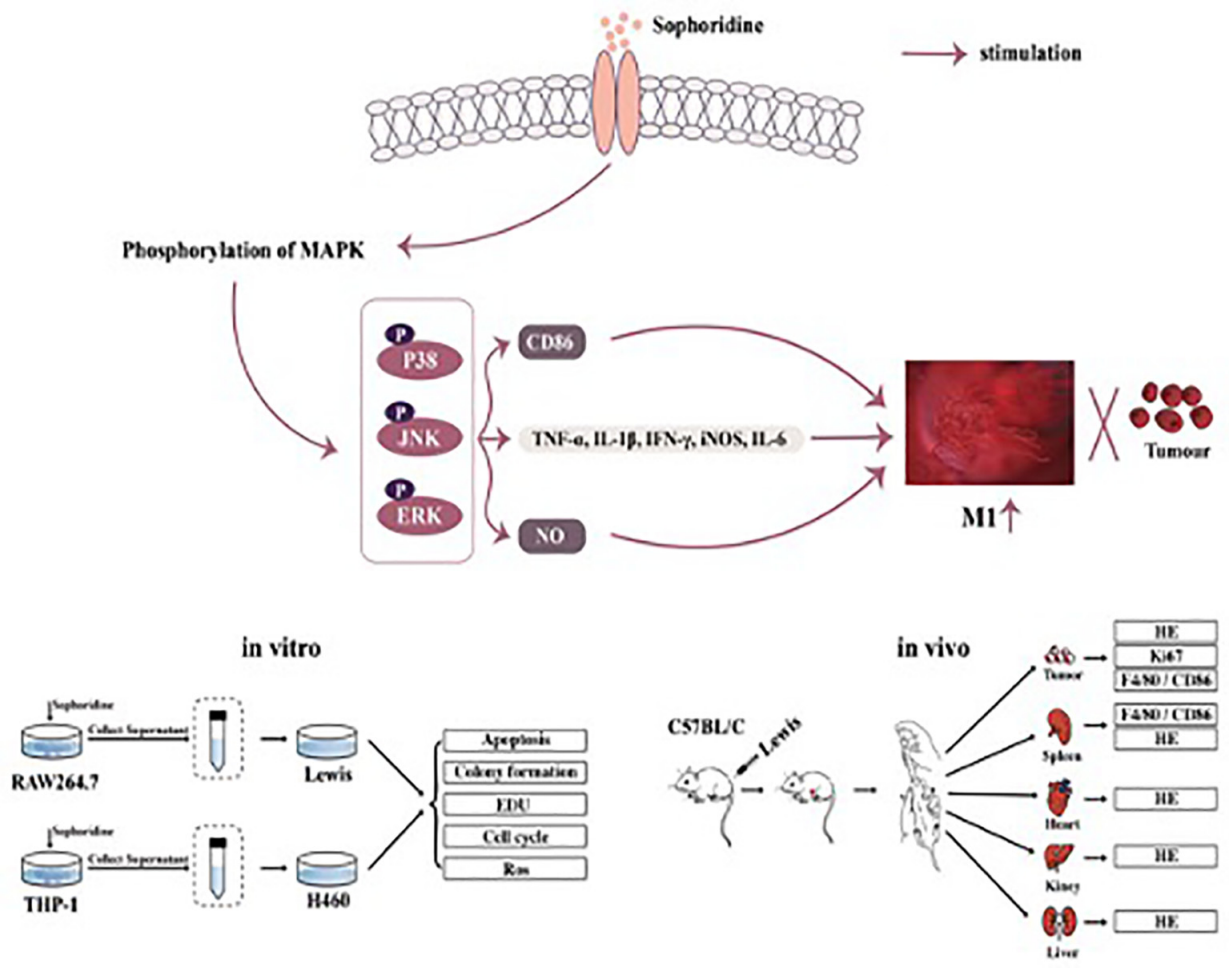
Sophoridine induced macrophages M1 polarisation *via* MAPKs pathway.

Herein, the regulatory effects of sophoridine on the differentiation of THP-1 cells into macrophages and the anti-tumour effects of sophoridine-stimulated M1 polarised macrophages towards lung cancer cells were investigated both *in vitro* and *in vivo*. Surface markers and cytokine production were employed to confirm the TAM polarisation to pro-inflammatory M1 status. The findings in this study clearly demonstrated that sophoridine was capable of promoting the polarisation of RAW264.7 and THP-1 cells into M1-like macrophages. The anti-tumour effects of sophoridine-stimulated M1 polarised macrophages were evaluated *via* co-culturing with lung cell lines (H460 and Lewis lung cancer cells). In addition, the *in vivo* anticancer effects of sophoridine were investigated in a subcutaneous xenograft tumour mice model (**Figure 2**).

## Materials and Methods

### Reagents and Antibodies

Sophoridine powder was purchased from MCE (purity > 98%). Anti-human/mouse CD86 PE, anti-human/mouse CD11b FITC, anti-mouse F4/80 FITC were purchased from BioLegend (CA, USA). Antibodies against total and phosphorylated MAPK, Ki67, F4/80 and CD86, and GAPDH were purchased from Cell Signaling Technology (MA, USA). SP600125 were purchased from MCE (NJ, USA). LPS and Phorbol 12-myristate 13-acetate (PMA) was obtained from Sigma-Aldrich (MO, USA).

### Cell lines and Cell Culture

The human and mouse lung cancer cell lines, H460 and Lewis, the human monocyte cell line, THP-1, and the mouse macrophages, RAW264.7, were kindly provided by the Chinese Academy of Sciences, China (Shanghai, China). Lewis and RAW264.7 cells were cultured in DMEM medium (Gibco, NY, USA), while H460 and THP-1 cells were cultured in RPMI-1640 medium (Gibco). All cultures were supplemented with 10% fetal bovine serum (Gibco) and 100 U/ml penicillin–streptomycin (Sigma-Aldrich). The cultures were kept under 5% CO_2_ at 37°C. THP-1 was differentiated into macrophages-like phenotype by stimulating with 150 nM PMA. The macrophages were polarised into M1 by 1 μg/ml LPS for 24 h. M0 macrophages were stimulated with 20 or 40 μg/ml sophoridine for 24 h. Afterwards, the supernatant of the treated cells was collected, and co-cultured with Lewis and H460, respectively.

### Conditioned Medium Preparation

Cell supernatants of macrophages were harvested after an additional 24 h of culture. The supernatants were centrifugated at 2,000 rpm for 10 min, and filtrated through a 0.22 µm cell strainer. Supernatants were collected as conditioned medium and stored at −80°C.

### CCK-8 Assays

After stimulating with different concentrations of sophoridine, the cell viability was detected using CCK-8 assay (Dojindo, Kumamato, Japan). The cells were seeded in 96-well flat plates at a density of 5 × 10^3^ cell/ml. After the indicated incubation time, 10 μl of CCK-8 reagent was added into each well. Cells were cultured for 1.5 h. The OD value of each well was detected at 450 nm. Cell viability rate (%) = OD450(test)/OD450 (control) × 100%.

### Flow Cytometry

After 24 h incubation, cells were washed with staining buffer (BioLegend. CA, USA), then incubated with anti-human/mouse PE-CD86 or human/mouse FITC-CD11b at room temperature for 20 min. Afterwards, the cells were collected, washed, and resuspended in cell staining buffer. Spleens were filtered through 40 μm nylon mesh strainer, and then were lysed for 5 min by using 1× Red Blood Cell Lysis Buffer. Cells were double stained with mouse FITC-CD11b and PE-CD86 fluorescently tagged antibodies. All data were detected by a CytExpert flow cytometer system and analysed by FlowJo software.

### RT-PCR analysis

Total RNA was isolated with Trizol (Sigma-Aldrich) method. cDNA was synthesised using the PrimeScript II 1st Strand cDNA Synthesis Kit (Takara, Tokyo, Japan). The mRNA expression was prepared by RT-PCR with SYBR Green PC Master Mix (Applied Biosystems, USA). The primer sets used were as follows: Mouse: IL-6: forward: ATAGGTGGACTGGACTCCCGA, reverse: TTTGGTGCTTCACAATTCAG; TNF-α: forward: GCGACGTGGAACTGGCAGAAG, reverse: GCCACAAGCAGGAATGAGAAGAGG; IL-1β: forward: ATGGCAATGTTCCTGAACTCAACT, reverse: CAGGACAGGTATAGATTCTTTCCTTT; iNOS: forward: GGACCCAGTGCCCTGCTTT, reverse: CACCAAGCTCATGCGGCCT; *β*-actin: forward: TGGAATCCTGTGGCATCCATGAAAC, reverse: TAAAACGCAGCTCAGTAACAGTCCG; Human: IL-1β: forward: TGCTCAAGTGTCTGAAGCAG, reverse: TGGTGGTCGGAGATTCGTAG; TNF-α: forward: CCCAGGGACCTCTCTCTAATC, reverse: ATGGGCTACAGGCTTGTCACT; IL-6: forward: ACCCTGACCATCCAAGTCAAA, reverse: TTGGCCTCGCATCTTAGAAAG; iNOS: forward: TGGAGCCAGTTGTGGATTGTC, reverse: GGTCGTAATGTCCAGGAAGTAG; GAPDH: forward: CACCAACTGCTTAGCACCCC, reverse: TGGTCATGAGTCCTTCCACG. Thermocycler conditions: initial holding at 50°C for 2 min; 95°C for 5 min and 15 s; 60°C for 15 s; 72°C for 30 s for 40 cycle sandals; holding at 72°C for 5 s. Reactions were conducted using an ABI PRISM^®^ 7,300 Sequence Detection System (Applied Biosystems, Canada). The results were expressed as RQ = 2− ΔΔCt.

### Griess Test

20 and 40 μg/ml sophoridine or 1 μg/ml LPS was added for 24 h. After the incubation, cell supernatants were collected for the measurement of Nitric Oxide (NO) by using NO assay kit (Sigma-Aldrich), and incubated for 10 min. A microplate reader was used to detect the OD value at 540 nm.

### Western Blot Analysis

Proteins (10 μg/lane) was dissolved in sodium dodecyl sulfate (SDS)-polyacrylamide gels and transferred onto a polyvinyl difluoride (PVDF) membrane (Millipore), then incubated overnight with primary antibody at 4°C. The blots were incubated with secondary antibodies for 2 h. The results were analysed using ImageJ software (National Institutes of Health, Maryland, USA).

### Cell apoptosis Assay

Cells were collected after 24 h incubation. The cell apoptosis was analysed using an Annexin V-FITC/PI Apoptosis Detection Kit (V13241) (Thermo, MA, USA). All samples were detected using a CytExpert flow cytometer (Beckman Coulter, USA).

### Colony Formation, Cell Cycle, and Cell Proliferation Analysis

Cells were seeded in 12-well flat plates at a density of 900 cells/well and incubated for 10 d. Cells were fixed with 4% paraformaldehyde (Beyotime, Shanghai, China) for 15 min and stained with crystal violet (Beyotime) for 15 min. Glacial acetic acid was added after taking pictures; the OD value of each well was detected at 590 nm. For cell cycle assay, the cells were stimulated with sophoridine (20 and 40 μg/ml) for 24 h. Cells were fixed with 70% ethanol at −20°C overnight. DNA content was detected by PI staining. Cell proliferation was measured using the 5-ethynyl-2′-deoxyuridine (EdU) staining. Cells (1 × 10^5^/ml) were incubated with 1 µM EdU solution (Thermo Fisher Scientific, Inc.) for 2 h, and then stained with 1× Hoechst 33342 solution (Beyotime) for 10 min at 25°C. The morphologic changes were observed using a fluorescence microscope (magnification, ×100).

### Measuring of Intracellular Reactive Oxygen Species

To measure the intracellular ROS level, the diluted DCFH-DA was added to the incubated cells for 30 min at 25°C in the dark. The intensity of fluorescence was detected using a CytExpert flow cytometer.

### Lewis Lung Cancer Tumour Xenograft Mice Experiment

Female C57BL/6J mice (6-week-old) were conducted in compliance with the relevant laws and institutional guidelines. The animal studies were approved by the Animal Care and Use Committee of Shanghai Institute of Food and Drug Control (the approval No. SIFDC18096). LLC cells (8 × 10^5^ cells) in 0.2 ml of PBS were injected subcutaneously into the right back side of the mouse. After 1 d, mice were intragastric (i.g.) administrated with 0.2 ml of sophoridine (15 mg/kg) and 0.2 ml of sophoridine (25 mg/kg) for 25 d.

### Haematoxylin–Eosin and Immunohistochemistry Staining

Tissue (heart, liver, kidney, and spleen) slides were stained with H&E. Slides were observed using a microscope (×100 magnification) for six fields. The expression of Ki67 was quantitatively evaluated using a fluorescence microscope (×100 magnification).

### Immunofluorescent Staining

To analyse the expression of M1-like macrophages in tumour tissues, tumour tissues were fixed by 4% paraformaldehyde for 10 min. Then, all tissues made into paraffin were cut at a thickness of 4 μm. For immunofluorescence, the primary antibodies were employed: F4/80 (1:500) and CD86 (1:500). For morphometric evaluation, five optical fields/tumour section were randomly chosen and imaged by a fluorescence microscope.

### Statistical Analysis

Values are expressed in Mean ± Standard Deviation of three independent experiments. GraphPad Prism 8.0 software (San Diego, CA, USA) was employed to analyse data. The difference between groups was measured using Unpaired Student’s t-test (two-tailed) and one-way ANOVA test. Values with *p <*0.05 (*) mean statistically different.

## Results

### Sophoridine Promoted Macrophages Switching to M1 Phenotype

The effects of sophoridine on cell viability of RAW 264.7 and THP-1-derived macrophages were measured by CCK-8 assay. As shown in **Figures 1B, C**, 10, 20, and 40 μg/ml sophoridine displayed negligible effects on cell viability compared to the untreated control groups (*p* > 0.05). Herein, 20 and 40 μg/ml sophoridine were used for subsequent experiments.

To determine whether sophoridine induced M1 macrophages polarisation, CD86, a co-stimulatory molecule, which is a differentiated marker of macrophages, was detected by flow cytometry analysis. As shown in **Figures 1D, E**, stimulated with LPS alone induced macrophages polarising to M1 phenotype, and the expressions of CD86 of RAW 264.7 and THP-1 macrophages were more than 80%. When treated with 20 or 40 μg/ml sophoridine, the expressions of CD86 marker of macrophages increased significantly compared to the control cells (*p* < 0.05). Moreover, THP-1 cells stimulated by sophoridine showed an approximately two to three-fold increase in the expression of CD86 surface marker relative to PMA-differentiated macrophages.

To further evaluate the effects of sophoridine on the release of the classic cytokines of M1-like macrophage, RT-PCR was carried out to detect the expression of IFN-*γ*, IL-1β, TNF-α, IL-6, and iNOS of RAW264.7 and THP-1-derived macrophages. As shown in **Figure 1F**, upon exposure to LPS or sophoridine, RAW264.7 produced noticeably greater amounts of iNOS compared to the untreated cells, followed by IFN-*γ* and TNF-α. **Figure 1G** presents that LPS- or sophoridine-stimulated THP-1-derived macrophages produced higher levels of pro-inflammatory cytokines of M1-like macrophage compared to the control cells (*p* < 0.05). Sophoridine induced the cytokine production of M1 macrophages in a.

In order to further determine whether sophoridine could up-regulate pro-inflammatory cytokines of M1 macrophages, the protein expressions of TNF-α and IL-1β were also detected by Western blotting. As shown in **Figures 1J, K**, LPS and sophoridine up-regulated the expression of TNF-α and IL-1β in both RAW264.7 and THP-1-derived macrophages. The results were highly consistent with the mRNA expression of TNF-α and IL-1β.

The results of mRNA expression of iNOS in macrophages (**Figures 1F, G** **)** showed that sophoridine increased the mRNA expression of iNOS in RAW264.7 and THP-1-derived macrophages compared to the control cells. NO production was further quantified by Griess in this study, and the effects of sophoridine on the release of NO in RAW264.7 and THP-1-derived macrophages were depicted in **Figures 1H, I**. LPS-stimulated RAW264.7 produced 22.5 μmoL/L NO, while 20 and 40 μg/ml sophoridine-stimulated RAW264.7 released 16 and 18 μmoL/L NO, respectively, which were up to 16- to 18-fold higher than the control level (*p* < 0.05). The level of NO production was also consistent with the mRNA expression of iNOS.

To determine whether sophoridine inhibits IL-4 and IL-13-induced RAW264.7 macrophage M2 polarisation, we detected the expression of the surface markers CD206 by flow cytometry in M2 macrophages after treatment with sophoridine. As shown in **Supplementary Figure 1**, significant up-regulation of CD206 was observed when RAW24.7 monocytes were treated with IL-4 and IL-13 for 24 h, and this was greatly reduced by sophoridine.

### MAPK Signalling Pathway Regulated the Sophoridine-Induced Differentiation and the Production of Pro-Inflammatory Cytokines

**Figure 3:**
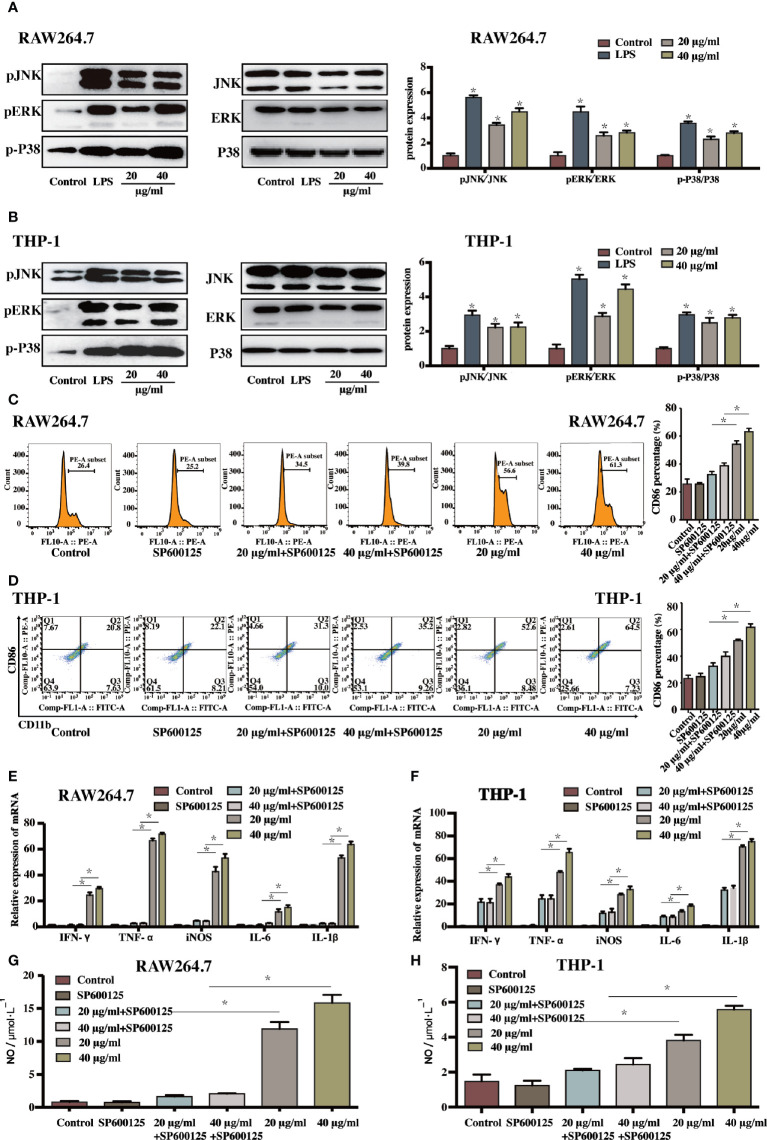
The sophoridine induced macrophages differentiation and produced pro-inflammatory cytokines *via* the MAPKs signaling pathway **(A, B)** The relative protein expression of p-JNK, p-ERK, and p-P38 (relative to JNK, ERK, and P38, respectively) in RAW264.7 and THP-1-derived macrophages determine by Western Blotting. **(C, D)** The percentage of CD86 of sophoridine-stimulated RAW264.7 and THP-1-derived macrophages with or without SP600125 treatment. **(E, F)** The relative expression of IFN-*γ*, TNF-α, IL-6, iNOS, and IL-1β mRNA in sophoridine-stimulated RAW264.7 and THP-1-derived macrophages with or without SP600125 treatment determine by RT-PCR. **(G, H)** The NO production of sophoridine-stimulated RAW264.7 and THP-1-derived macrophages with or without SP600125 treatment (n = 3), *p* < 0.05 (*).

As macrophage polarisation needs the activation of unique transcription factors; the potential signalling pathways involved in sophoridine-stimulated M1 macrophage polarisation need to be further elucidated. The MAPK signalling pathway, which has been proved to play an essential role in mediating inflammatory response (25), was carried out. Prior to the sophoridine stimulation, a JNK inhibitor, SP600125, was employed to evaluate its effect on MAPK signalling pathway. SP600125 down-regulated the expression of p-JNK of RAW246.7 and THP-1-derived macrophages compared to untreated cells (**Supplementary Figure 1**). Hence, SP600125 blocked the MAPK signalling pathway *via* inactivation of p-JNK. Afterwards, the differentiated macrophages were stimulated by 20 and 40 μg/ml sophoridine to determine whether sophoridine can induce the M1 macrophages polarisation. According to the **Figures 3A, B**, LPS and sophoridine (20 and 40 μg/ml) triggered the phosphorylation of JNK, ERK, and p38 MAPK in RAW264.7 and THP-1-derived macrophages. RAW264.7 and THP-1 cells were pre-treated with SP600125, and then stimulated with or without sophoridine (20 and 40 μg/ml). The expression of surface markers, CD86 and CD11b (**Figures 3C, D** **)** as well as pro-inflammatory cytokines, including IL-1β, IFN-*γ*, TNF-α, iNOS, and IL-6 (**Figures 3E, F** **)** in macrophages were detected by flow cytometry and RT-PCR analysis, respectively. The expression of CD86 and the pro-inflammatory cytokines in RAW264.7 and THP-1-derived macrophages was partly prevented by SP600125. Although RAW264.7 and THP-1-derived macrophages were co-stimulated with 20/40 μg/ml sophoridine, the mRNA expressions of IFN-*γ*, TNF-α, IL-6, iNOS, and IL-1β were significantly lower than that of stimulated with sophoridine alone (p < 0.05). In addition, pre-treatment with SP600125 down-regulated the expression of CD86 in sophoridine-stimulated macrophages when compared with the macrophages without pre-treatment with SP600125. Besides, the expression of NO in RAW264.7 and THP-1-derived macrophages was measured. After pre-treating with SP600125 on sophoridine-stimulated RAW264.7 and THP-1-derived macrophages, the NO production was considerably lower than the macrophages without treatment with SP600125 (**Figures 3G, H**, p < 0.05). This result was consistent with the down-regulated mRNA expression of iNOS.

### Sophoridine-Stimulated Macrophage-Lung Cancer Cell Crosstalk Induced Cell Apoptosis

The supernatants of sophoridine-treated RAW264.7 and THP-1-derived macrophages were collected and co-cultured with H460 and Lewis lung cancer cells, respectively. Cell colony formation, cell apoptosis, and distribution of the cell cycle were conducted on H460 and Lewis cells. Prior to these analyses, H460 and Lewis cells were stimulated with various concentrations of sophoridine alone for 48 h. The results show that the IC50 for H460 and Lewis was 73.49 and 64.95 μg/ml at 24 h and 53.52 and 40.10 μg/ml at 48 h, respectively (**Supplementary Figure 2**), demonstrating that sophoridine inhibited the growth of H460 and Lewis cells.

**Figure 4:**
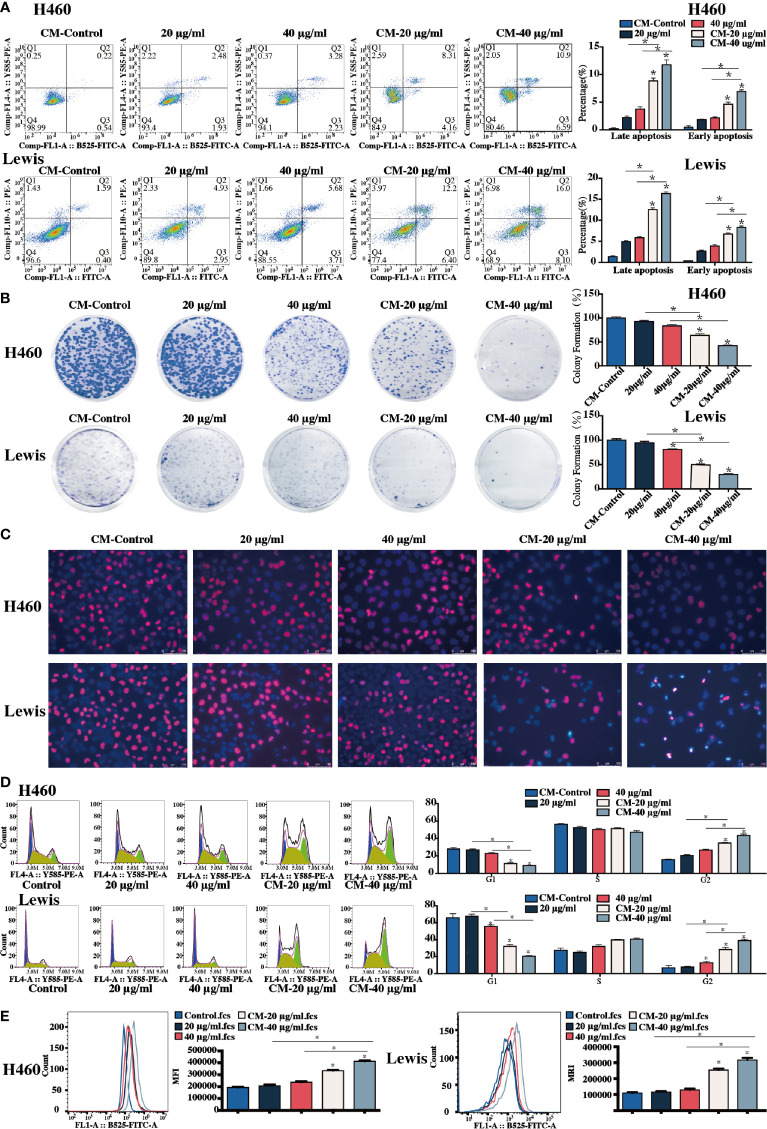
Sophoridine-stimulated macrophage-lung cancer cell crosstalk induced cell apoptosis, cell colony formation, and suppressed cell proliferation. **(A)** The cell apoptosis of H460 with or without infiltration of sophoridine-stimulated THP-1-derived macrophages, and Lewis lung cancer cells with or without sophoridine-stimulated RAW264.7 macrophages determine by flow cytometry analysis. **(B)** The cell colony formation of H460 with or without infiltration of sophoridine-stimulated THP-1-derived macrophages, and Lewis lung cancer cells with or without sophoridine-stimulated RAW264.7 macrophages. **(C)** The cell proliferation and apoptosis of H460 with or without infiltration of sophoridine-stimulated THP-1-derived macrophages, and Lewis lung cancer cells with or without sophoridine-stimulated RAW264.7 macrophages determine by EdU and Hoechst staining. **(D)** The percentage of cell number in the G1, S, and G2/M phase during the cell cycle of H460 with or without infiltration of sophoridine-stimulated THP-1-derived macrophages, and Lewis lung cancer cells with or without sophoridine-stimulated RAW264.7 macrophages detect by flow cytometry using PI staining. **(E)** ROS intensity with or without infiltration of sophoridine-stimulated macrophages (n = 3), *p* < 0.05 (*).

The apoptotic rate of H460 and Lewis cells was measured. Infiltration of H460 and Lewis lung cancer cells with sophoridine-induced RAW264.7 and THP-1-derived macrophages, respectively, significantly increased the percentages of the later and early apoptotic rate of lung cancer cells compared to the untreated and sophoridine-treated alone H460 and Lewis cells (*p* < 0.05) (**Figure 4A**). These results suggested that 20 and 40 μg/ml sophoridine-stimulated macrophages effectively induced the cell apoptosis of H460 and Lewis cells. Quantitative analysis of colony formation in **Figure 4B** demonstrates that the colony formations of macrophages infiltrated H460 and Lewis cancer cells were significantly lower than control and sophoridine-treated alone lung cancer cells (*p* < 0.05). This trend was in agreement with the results of cell apoptosis of H460 and Lewis cells, suggesting that sophoridine-stimulated macrophages significantly effectively inhibited cell colony forming activities of H460 and Lewis lung cancer cells at the doses of 20 and 40 μg/ml.

Morphologic changes and cell proliferation of lung cancer cells were detected after the Hoechst and EdU staining. The obvious apoptotic features, such as nuclear shrinkage, irregular condensation of chromatin, and apoptotic bodies, were detected in sophoridine stimulated-H460 and Lewis cells (**Figure 4C**, blue staining). In addition, the area of the red staining, representing the cell proliferation, decreased dramatically in macrophages infiltrated H460 and Lewis cells compared to untreated cells. Moreover, this phenomenon seemed to be more obvious compared to those in Lewis lung cancer cells. These results revealed that sophoridine-stimulated macrophages at the doses of 20 and 40 μg/ml induced the cell apoptosis and suppressed the cell proliferation of lung cancer cells.

**Figure 4D** shows the percentage of cell number in each phase of cell cycle of H460 and Lewis cells. The percentage of THP-1-infiltrated H460 cells in the G2/M phase significantly increased, whilst the percentage of RAW264.7-infiltrated Lewis cells in the S and G2/M phases increased when compared with the untreated cells (*p* < 0.05). The arrest effect in macrophage-infiltrated cells was more significant than in lung cancer cells stimulated with sophoridine alone (*p* < 0.05). These results suggested that sophoridine-stimulated THP-1-derived arrested the cell cycle of H460 in the G2/M phase, while sophoridine-stimulated RAW264.7 macrophages arrested the cell cycle of Lewis cells in the S phase partially and the G2/M phase.

The ROS generation in two lung cancer cells was detected by flow cytometry analysis, and the results are shown in **Figure 4E**. The ROS generation increased considerably in sophoridine-stimulated macrophages infiltrated compared to untreated cells (*p* < 0.05). M1 polarised macrophages infiltrated lung cancer cells generated greater ROS than sophoridine-administrated.

### Sophoridine Inhibited Tumour Growth *In Vivo*

**Figure 5:**
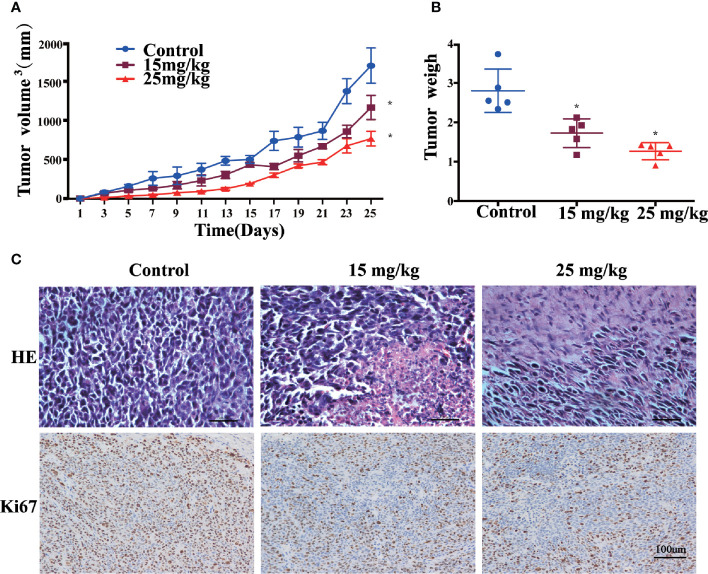
Sophoridine inhibited tumour growth of Lewis-bearing mice and promoted M1 polarisation of macrophages *in vivo*. **(A)** Administration of 15 and 25 mg/kg sophoridine decreased the tumour volume (mm^3^). **(B)** Administration of 15 and 25 mg/kg sophoridine decreased the tumour weigh. **(C)** Administration of 15 and 25 mg/kg sophoridine decreased the infiltration of inflammatory cells measured by H&E staining, and the expression of Ki67 measured by IHC staining, in tumour tissues from Lewis-bearing mice p < 0.05 (*).

**Figures 5A, B** present that oral administration of sophoridine at doses of 15 and 25 mg/kg significantly suppressed tumour growth markedly, which was reflected by the decrease of the volume and the weight of tumour, and the final volume and the weight of tumour from mice administrated with sophoridine were significantly lower than those from control mice (p < 0.05). Consistent with the results in **Figure 5C**, 15 and 25 mg/kg sophoridine induced the destruction of architectures of tumour tissues, and the infiltration of large number of inflammatory cells into tumour tissues reflected by H&E staining.

The IHC staining of Ki67 (**Figure 5C**) presents that the tumour tissues from mice administrated with sophoridine had less positive staining compared to the untreated mice (p < 0.05), indicating that administration of sophoridine at the doses of 15 and 25 mg/kg significantly inhibited the expression of Ki67 in tumour tissues. These pathological analyses indicated that the administration of sophoridine effectively prevented the tumour growth.

### Sophoridine Promoted M1 Polarisation of Macrophages *In Vivo*

**Figure 6:**
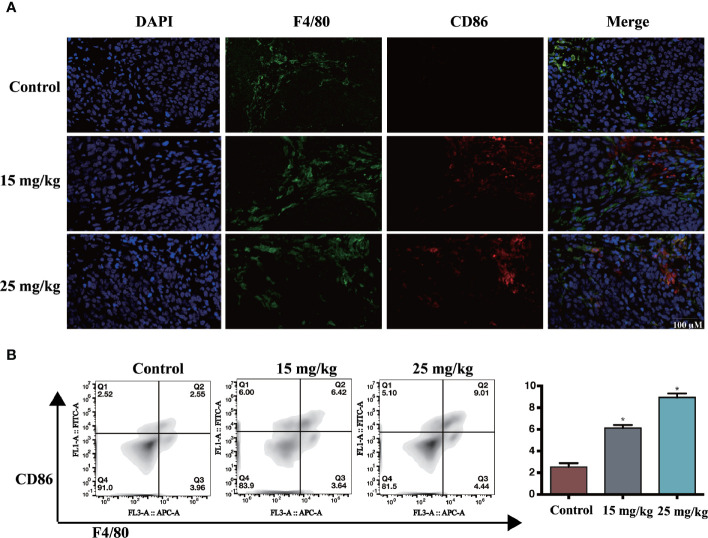
**(A)** The expression of CD86 and F4/80, determined by IHC. Scale bar: 50 μm **(B)** Administration of 15 and 25 mg/kg sophoridine increased the expression of the ratio of CD86/F4/80 (n = 3), *p* < 0.05 (*).

The lung sections from different groups were double stained with the M1-marker CD86 and the macrophage marker F4/80. The results are shown in **Figure 6A**; sophoridine increased the percentage of CD86 significantly. Flow cytometry analysis was also employed to measure the ratio of CD86 to F4/80. The result of spleen in **Figure 6B** was consistent with the staining results, presenting that sophoridine increased the ratio of CD86/F4/80.

### Sophoridine Had No Toxicity Toward Heart/Kidney/Spleen/Liver

**Figure 7:**
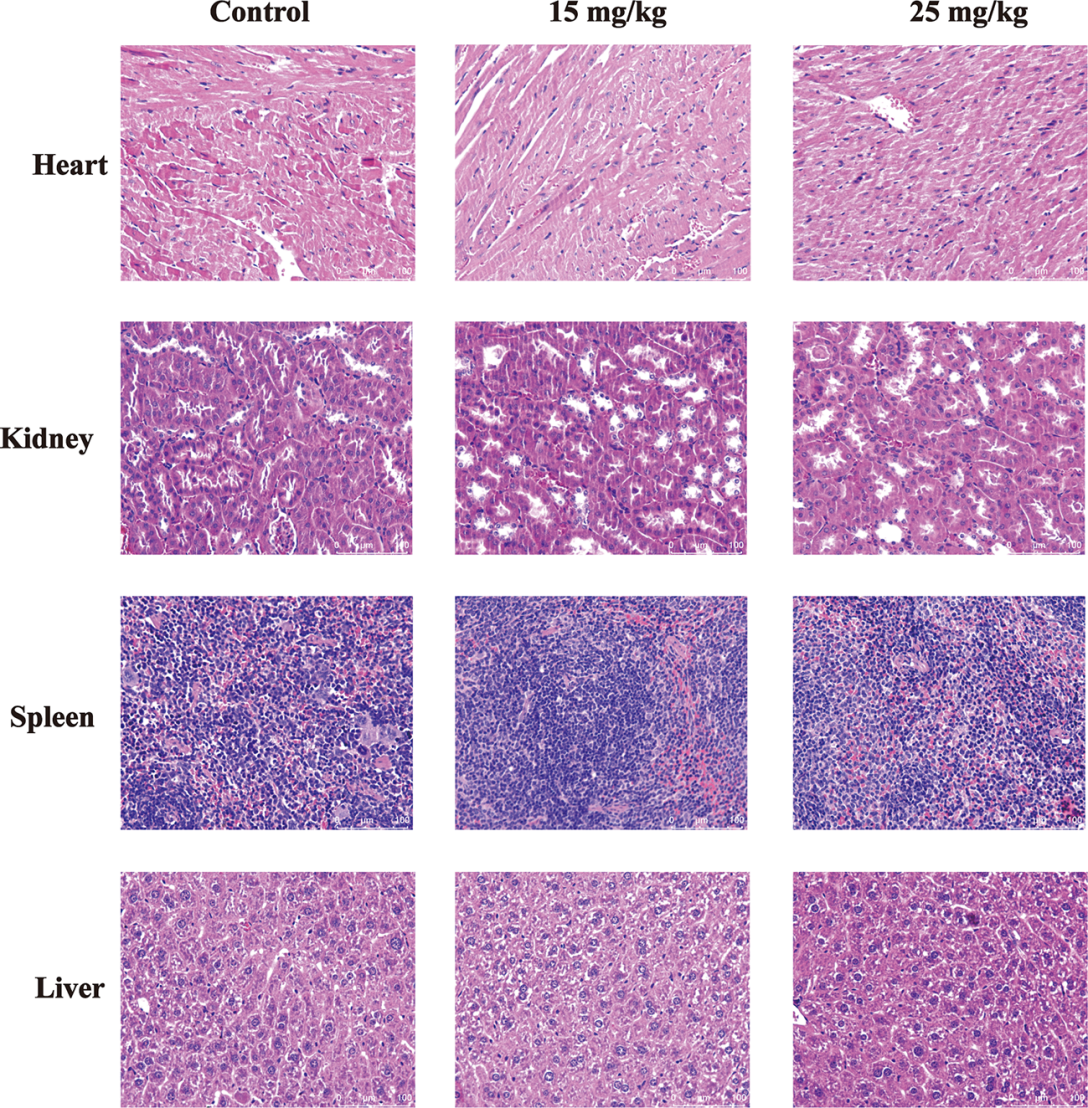
The toxicity of sophoridine on the heart, liver, kidney, and spleen tissues *in vivo*. The sections were stained with H&E staining. The pictures are representative from control and 15 and 25 mg/kg sophoridine groups.

The *in vivo* toxicity of sophoridine was evaluated in mice. Following oral administration of sophoridine at the doses of 15 and 25 mg/kg for 25 days, the systemic toxicity was evaluated by H&E staining. As shown in **Figure 7**, there was no obvious difference between control groups and sophoridine-administration groups. The tissues collected from mice were stained with H&E to further monitor the cardiac, liver, spleen, and kidney toxicity after the oral administration. The histological structure of the heart, kidney, liver, and spleen was observed and compared microscopically. There was no obvious histological change after oral administration of sophoridine. These findings revealed that sophoridine was an effective agent, which suppressed the xenograft lung tumour growth *in vivo*, with well-tolerated toxicity.

## Discussion

Monocyte-to-macrophage differentiation is a vital stage during the onset of immune responses. THP-1 cell has been widely employed to investigate the function activity and the differentiation of monocyte and macrophage *in vitro* (26). PMA is considered as the most effective stimulating agent to induce the differentiation of THP-1 cells, which can represent a simplified macrophage model (27). In this study, PMA was employed to induce the differentiation of THP-1 cells to macrophages, and PMA-stimulated THP-1-derived macrophages were used for subsequent experiments.

CD86, a co-stimulatory molecule, which is the differentiated marker of macrophages, was detected in this study. The up-regulated expression of CD86 surface marker revealed that sophoridine stimulated RAW264.7 and THP-1 cells to differentiate into macrophage-like cells and might induce macrophages polarising to M1 phenotype. M1 phenotype produces pro-inflammatory cytokines and plays a critical role in microbial and tumour regression, while M2 expresses anti-inflammatory factors and participants in the immune regulation and tumour progression. In the tumour microenvironment, the TAMs adopt an immunosuppressive M2-like phenotype important to promote cancer growth. To further evaluate the influences of sophoridine on the production of the typical cytokines of M1 macrophage, the expression of IFN-γ, TNF-α, IL-6, IL-1β, and iNOS was measured. The results indicated that sophoridine induced the cytokines production of M1 macrophages. Pathogens, in particular of those containing LPS, induce chronic inflammation (28), which is related to the increase in NO production. Macrophages appear to be the major cellular source of NO (29). Previous studies have demonstrated LPS-stimulated macrophages produced high level of NO, thus leading to the macrophage’s differentiation. iNOS is the primary enzyme and makes the great contributions to the NO production in inflammatory processes (30). A change in iNOS activity directly affects the production of NO (31). This study demonstrated that sophoridine induced the activation of iNOS activity of macrophages to produce NO, thus leading to the M1-macrophages polarisation. Macrophage activation state is vital to regulate between inflammation and resolution or tissue homeostasis and disease pathogenesis. Moreover, it was demonstrated that sophoridine inhibit skewing of M2-like macrophages (**Supplementary Figure 3**). However, we need more experiments to verify that sophoridine inhibits M2-like macrophages.

MAPK signalling pathway has been reported to be associated with macrophage activation and reprogramme TAMs towards M1 macrophages polarisation (13, 14). SP600125 was used to block the initiation of signal transduction (32), and the results suggested that stimulation of JNK inhibitor before polarising with LPS or sophoridine blocked the induction of the M1 marker CD86 and decreased the production of the M1 cytokines in sophoridine-stimulated macrophages. In addition, iNOS activity and NO production were drastically blocked by JNK inhibitor, which resulted in the inhibition of iNOS activity, thus leading to the reduction of NO production in sophoridine-stimulated RAW264.7 and THP-1-derived macrophages. Taken together, these results revealed that JNK acted as an initiator for activation of MAPKs signalling pathway during the process of M1-like macrophage polarisation. However, there are still some limitations in this study. On the one hand, this study focussed primarily on the MAPK signalling pathway, despite any other potential mechanisms involved in the macrophage polarisation. On the other hand, only CD86 was employed as the surface marker to discriminate macrophages. More definitive biochemical markers to differentiate macrophage populations are needed.

The interactions of lung cancer cells with the RAW264.7/THP-1-derived macrophages are bidirectional. RAW264.7 and THP-1-derived macrophages seem to be educated by the lung cancer cells to lead to their activation under the co-culture conditions (33).

ROS generation plays a key role in several signalling pathways. Elevated ROS in cancer cells promotes the cell apoptosis in response to the cellular stress induced by chemotherapy (34). Varieties of drugs have been reported to exert their effects *via* the activation of induced the cell apoptosis through ROS generation. ROS are the natural products of cellular metabolism (35). Herein, infiltration with sophoridine-stimulated macrophages induced the cell apoptosis and ROS generation of lung cancer cells, indicating that ROS production was an upstream regulator of sophoridine inducing lung cancer cell apoptosis.

Sophoridine-stimulated macrophages arrested the cell cycle of Lewis cells in the S phase partially as well as in the G2/M phase which prevented DNA from replicating properly, and thus inhibited the tumour growth. This result was agreement with a previous study (21), reporting that sophoridine arrested the S phase of pancreatic cancer cell cycle. The damage and error induced by the cell cycle arrest, which occurs in the course of cell division, are difficult to repair (36). Herein, sophoridine-stimulated macrophages induced the DNA damage and cell cycle arrest, leading to the suppression of lung cancer cell proliferation. Large numbers of macrophages infiltrated into lung adenocarcinoma tissues were related to the poor patient prognosis (37). The data obtained in this study revealed that the RAW264.7 and THP-1-derived macrophages likely underwent differentiation into M1-like macrophage, thus exerted biological effects towards lung cancer cells. Therefore, RAW264.7 and THP-1-derived macrophages and lung cancer cells altered the behaviors mutually between each other. Furthermore, lung cancer cells educated by macrophages acquired myeloid features, including the properties of cell colony formation, cell apoptosis, and cell proliferation.

High proliferation rate is a characteristic of cancer. Ki67 protein is well characterised at the molecular level and is normally used as a prognostic and predictive marker for the diagnosis and treatment of cancers (38). Ki67 is rapidly degraded with a half-life of 1–1.5 h. This makes the Ki-67 antigen an outstanding marker for the detection of the cell proliferation in normal and tumour cell populations (39). Therefore, it deserves further investigation and development, such as testing in more sophisticated *in vitro* and appropriate *in vivo* models. Cellular proliferation can be identified by different methods, IHC staining for the Ki-67 antigen has become widely used in histopathology, especially as a proliferator in various tumour types. High expression level of Ki-67 in the tumour tissue has been reported to be associated with poor prognosis in NSCLC (38). However, a study reported that Ki-67 level did not affect the survival rate of cancer patients (40). In this study, after administration of sophoridine, the expression of Ki67 in tumour tissues decreased considerably compared to untreated mice. These results confirmed that sophoridine inhibited the cell proliferation and demonstrated a favorable anti-tumour effect *in vivo*.

Macrophages are the most prominent component of leukocytes that infiltrate tumour-bearing mice and humans with various types of cancers (41). Considering the influence of sophoridine on tumour growth, the sophoridine-mediated effects on altered TAM polarisation in tumour tissues *in vivo* were investigated. These observations link TAMs highlighted the anti-tumourigenic effect of sophoridine, specifically *via* the MAPK signalling pathway, provided a promising strategy to modulate the environment of lung cancer cells and gave scientific support for the clinical application of sophoridine.

## Data Availability Statement

The original contributions presented in the study are included in the article/**Supplementary Material**. Further inquiries can be directed to the corresponding authors.

## Ethics Statement

The animal study was reviewed and approved by the Animal Care and Use Committee of Shanghai Institute of Food and Drug Control.

## Author Contributions

GG, TL and BZ conceived and designed the study. BZ, XH, HZ,YY, JH and QT performed the experiments. BZ and XH wrote the paper. GG and TL reviewed and edited the manuscript. All authors contributed to the article and approved the submitted version.

## Funding

This work was financially supported by the grants of NSF of China (81922070, 81973286, 81773687), the National Key Research and Development Program of China (2020YFC0845400, 2017YFC1700200, 2017YFC1702000), Program of Shanghai Academic/Technology Research Leader (18XD1403600), Shanghai Talent Development Fund (2019093), the Three-year Action Plan of Shanghai TCM Development (ZY-(2018-2020)-CCCX-5001), Natural Science Foundation of Shanghai (No.19ZR1447800), Shuguang Program (18SG40) supported by Shanghai Education Development Foundation and Shanghai Municipal Education Commission, The Health System Independent Innovation Science Foundation of Shanghai Putuo District (ptkwws201802), and Shanghai Science and Technology Commission Medical Guidance Project(19411972400).

## Conflict of Interest

The authors declare that the research was conducted in the absence of any commercial or financial relationships that could be construed as a potential conflict of interest.

## Supplementary Material

Supplementary Figure 1 SP600125 inhibited the expression of pJNK and JNK in macrophages.

Supplementary Figure 2 Sophoridine inhibited the growth of lung cancer cells in dose-dependent manners.

Supplementary Figure 3 Sophoridine suppressed M2 polarisation of macrophages *in vitro*.

## References

[1] Barta JA Powell C A Wisnivesky JP. Global Epidemiology of Lung Cancer. Ann Glob Health (2019) 85:8. 10.5334/aogh.2419 30741509 PMC6724220

[2] Siegel RL Miller K D Jemal A. Cancer statistics, 2020. CA Cancer J Clin (2020) 70:7–30. 10.3322/caac.21590 31912902

[3] Zappa C Mousa SA. Non-small cell lung cancer: current treatment and future advances. Transl Lung Cancer Res (2016) 5:288–300. 10.21037/tlcr.2016.06.07 27413711 PMC4931124

[4] Lawrenson R Lao C Brown L Moosa L Chepulis L Keenan R. Management of patients with early stage lung cancer – why do some patients not receive treatment with curative intent? BMC Cancer (2020) 20:109. 10.1186/s12885-020-6580-6 32041572 PMC7011272

[5] Baghban R Roshangar L Jahanban-Esfahlan R Seidi K Ebrahimi-Kalan A Jaymand M. Tumor microenvironment complexity and therapeutic implications at a glance. Cell Commun Signaling (2020) 18:59. 10.1186/s12964-020-0530-4 PMC7140346 32264958

[6] Hill BS Sarnella A D’Avino G Zannetti A. Recruitment of stromal cells into tumour microenvironment promote the metastatic spread of breast cancer. Semin Cancer Biol (2020) 60:202–13. 10.1016/j.semcancer.2019.07.028 31377307

[7] Epelman S Lavine K J Randolph GJ. Origin and functions of tissue macrophages. Immunity (2014) 41:21–35. 10.1016/j.immuni.2014.06.013 25035951 PMC4470379

[8] Parisi L Gini E Baci D Tremolati M Fanuli M Bassani B. Macrophage Polarization in Chronic Inflammatory Diseases: Killers or Builders? J Immunol Res (2018) 2018:8917804–8917804. 10.1155/2018/8917804 29507865 PMC5821995

[9] Atri C Guerfali F Z Laouini D. Role of Human Macrophage Polarization in Inflammation during Infectious Diseases. Int J Mol Sci (2018) 19:1801. 10.3390/ijms19061801 PMC6032107 29921749

[10] Arora S Dev K Agarwal B Das P Syed MA. Macrophages: Their role, activation and polarization in pulmonary diseases. Immunobiology (2018) 223:383–96. 10.1016/j.imbio.2017.11.001 PMC7114886 29146235

[11] Viola A Munari F Sánchez-Rodríguez R Scolaro T Castegna A. The Metabolic Signature of Macrophage Responses. Front Immunol (2019) 10:1462. 10.3389/fimmu.2019.01462 PMC6618143 31333642

[12] Gan Z-S Wang Q-Q Li J-H Wang X-L Wang Y-Z Du H-H. Iron Reduces M1 Macrophage Polarization in RAW264.7 Macrophages Associated with Inhibition of STAT1. Mediators Inflamm (2017) 2017:8570818. 10.1155/2017/8570818 28286378 PMC5327769

[13] Neamatallah T. Mitogen-Activated Protein Kinase Pathway: A Critical Regulator in Tumor-associated Macrophage Polarization. J Microsc Ultrastruct (2019) 7:53–6. 10.4103/JMAU.JMAU_68_18 PMC6585481 31293885

[14] Xu F Wei Y Tang Z Liu B Dong J. Tumor−associated macrophages in lung cancer: Friend or foe? (Review). Mol Med Rep (2020) 22:4107–15. 10.3892/mmr.2020.11518 PMC7533506 33000214

[15] Rizeq B Gupta I Ilesanmi J AlSafran M Rahman M M Ouhtit A. The Power of Phytochemicals Combination in Cancer Chemoprevention. J Cancer (2020) 11:4521–33. 10.7150/jca.34374 PMC7255361 32489469

[16] Zhang Y Xu G Zhang S Wang D Saravana Prabha P Zuo Z. Antitumor Research on Artemisinin and Its Bioactive Derivatives. Nat Prod Bioprospect (2018) 8:303–19. 10.1007/s13659-018-0162-1 PMC6102173 29633188

[17] Mushtaq S Abbasi BH Uzair B Abbasi R. Natural products as reservoirs of novel therapeutic agents. EXCLI J (2018) 17:420–51. 10.17179/excli2018-1174 PMC5962900 29805348

[18] ur Rashid H Rasool S Ali Y Khan K Martines MAU. Anti-cancer potential of sophoridine and its derivatives: Recent progress and future perspectives. Bioorg Chem (2020) 99:103863. 10.1016/j.bioorg.2020.103863 32334197

[19] Peng Z Guan Q Luo J Deng W Liu J Yan R. Sophoridine exerts tumor-suppressive activities via promoting ESRRG-mediated β-catenin degradation in gastric cancer. BMC Cancer (2020) 20:582. 10.1186/s12885-020-07067-x 32571331 PMC7310191

[20] Wang R Liu H Shao Y Wang K Yin S Qiu Y. Sophoridine Inhibits Human Colorectal Cancer Progression via Targeting MAPKAPK2. Mol Cancer Res (2019) 17:2469–79. 10.1158/1541-7786.MCR-19-0553 31575657

[21] Xu Z Zhang F Bai C Yao C Zhong H Zou C. Sophoridine induces apoptosis and S phase arrest via ROS-dependent JNK and ERK activation in human pancreatic cancer cells. J Exp Clin Cancer Res (2017) 36:124. 10.1186/s13046-017-0590-5 28893319 PMC5594456

[22] Wang WX Sun ZH Chen HM Xu B N Wang FY. Role and mechanism of Sophoridine on proliferation inhibition in human glioma U87MG cell line. Int J Clin Exp Med (2015) 8:464–71. 1940-5901/IJCEM0003106 PMC4358473 25785018

[23] Zhuang H Dai X Zhang X Mao Z Huang H. Sophoridine suppresses macrophage-mediated immunosuppression through TLR4/IRF3 pathway and subsequently upregulates CD8+ T cytotoxic function against gastric cancer. Biomed Pharmacother (2020) 121:109636. 10.1016/j.biopha.2019.109636 31733580

[24] Zhu L Huang S Li J Chen J Yao Y Li L. Sophoridine inhibits lung cancer cell growth and enhances cisplatin sensitivity through activation of the p53 and Hippo signaling pathways. Gene (2020) 742:144556. 10.1016/j.gene.2020.144556 32165304

[25] Du W Hu H Zhang J Bao G Chen R Quan R. The Mechanism of MAPK Signal Transduction Pathway Involved with Electroacupuncture Treatment for Different Diseases. Evid Based Complement Alternat Med (2019) 2019:8138017–8138017. 10.1155/2019/8138017 31467579 PMC6699341

[26] Smith SR Schaaf K Rajabalee N Wagner F Duverger A Kutsch O. The phosphatase PPM1A controls monocyte-to-macrophage differentiation. Sci Rep (2018) 8:902. 10.1038/s41598-017-18832-7 29343725 PMC5772551

[27] Chanput W Peters V Wichers H. THP-1 and U937 Cells. In: The Impact of Food Bioactives on Gut Health: In Vitro and Ex Vivo Models. Switzerland: Springer (2015). 147–59. 29787039

[28] Xue Q Yan Y Zhang R Xiong H. Regulation of iNOS on Immune Cells and Its Role in Diseases. Int J Mol Sci (2018) 19:3805. 10.3390/ijms19123805 PMC6320759 30501075

[29] Hirayama D Iida T Nakase H. The Phagocytic Function of Macrophage-Enforcing Innate Immunity and Tissue Homeostasis. Int J Mol Sci (2017) 19:92. 10.3390/ijms19010092 PMC5796042 29286292

[30] Hwang J-S Kwon M-Y Kim K-H Lee Y Lyoo IK Kim JE. Lipopolysaccharide (LPS)-stimulated iNOS Induction Is Increased by Glucosamine under Normal Glucose Conditions but Is Inhibited by Glucosamine under High Glucose Conditions in Macrophage Cells. J Biol Chem (2017) 292:1724–36. 10.1074/jbc.M116.737940 PMC5290947 27927986

[31] McNeill E Crabtree MJ Sahgal N Patel J Chuaiphichai S Iqbal AJ. Regulation of iNOS function and cellular redox state by macrophage Gch1 reveals specific requirements for tetrahydrobiopterin in NRF2 activation. Free Radic Biol Med (2015) 79:206–16. 10.1016/j.freeradbiomed.2014.10.575 PMC4344222 25451639

[32] Wu Q Wu W Jacevic V Franca TCC Wang X Kuca K. Selective inhibitors for JNK signalling: a potential targeted therapy in cancer. J Enzyme Inhib Med Chem (2020) 35:574–83. 10.1080/14756366.2020.1720013 PMC7034130 31994958

[33] Genin M Clement F Fattaccioli A Raes M Michiels C. M1 and M2 macrophages derived from THP-1 cells differentially modulate the response of cancer cells to etoposide. BMC Cancer (2015) 15:577–7. 10.1186/s12885-015-1546-9 PMC4545815 26253167

[34] Zhang J Lei W Chen X Wang S Qian W. Oxidative stress response induced by chemotherapy in leukemia treatment (Review). Mol Clin Oncol (2018) 8:391–9. 10.3892/mco.2018.1549 PMC5867396 29599981

[35] Yang H Villani RM Wang H Simpson MJ Roberts MS Tang M. The role of cellular reactive oxygen species in cancer chemotherapy. J Exp Clin Cancer Res (2018) 37:266. 10.1186/s13046-018-0909-x 30382874 PMC6211502

[36] Pan Z Zhang X Yu P Chen X Lu P Li M. Cinobufagin Induces Cell Cycle Arrest at the G2/M Phase and Promotes Apoptosis in Malignant Melanoma Cells. Front Oncol (2019) 9:853–3. 10.3389/fonc.2019.00853 PMC6738445 31552178

[37] Cao L Che X Qiu X Li Z Yang B Wang S. M2 macrophage infiltration into tumor islets leads to poor prognosis in non-small-cell lung cancer. Cancer Manag Res (2019) 11:6125–38. 10.2147/CMAR.S199832 PMC6613613 31308749

[38] Li LT Jiang G Chen Q Zheng JN. Ki67 is a promising molecular target in the diagnosis of cancer (Review). Mol Med Rep (2015) 11:1566–72. 10.3892/mmr.2014.2914 25384676

[39] Miller I Min M Yang C Tian C Gookin S Carter D. Ki67 is a Graded Rather than a Binary Marker of Proliferation versus Quiescence. Cell Rep (2018) 24:1105–1112.e5. 10.1016/j.celrep.2018.06.110 30067968 PMC6108547

[40] Wu Q Ma G Deng Y Luo W Zhao Y Li W. Prognostic Value of Ki-67 in Patients With Resected Triple-Negative Breast Cancer: A Meta-Analysis. Front Oncol (2019) 9:01068. 10.3389/fonc.2019.01068 PMC6811517 31681601

[41] Lin Y Xu J Lan H. Tumor-associated macrophages in tumor metastasis: biological roles and clinical therapeutic applications. J Hematol Oncol (2019) 12:76. 10.1186/s13045-019-0760-3 31300030 PMC6626377

