# Supplementary figures and images for: Sophoridine Inhibits the Tumour Growth of Non-Small Lung Cancer by Inducing Macrophages M1 Polarisation via MAPK-Mediated Inflammatory Pathway
Source: Front Oncol. 2021 Feb 24;11:634851. doi: 10.3389/fonc.2021.634851 (PMC7943889; doi:10.3389/fonc.2021.634851)

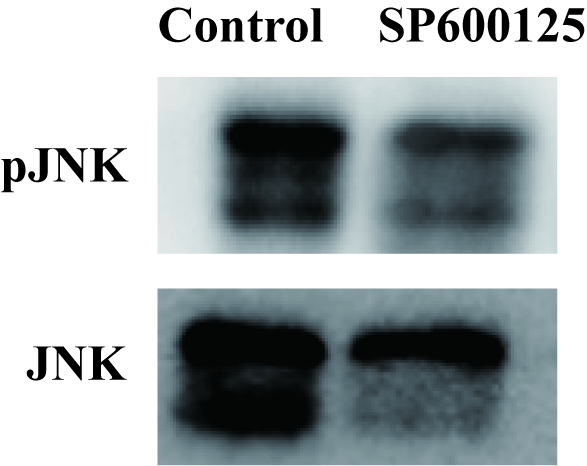

Supplement: Supplementary Figure 1 — SP600125 inhibited the expression of pJNK and JNK in macrophages. [file Image_1.tif]

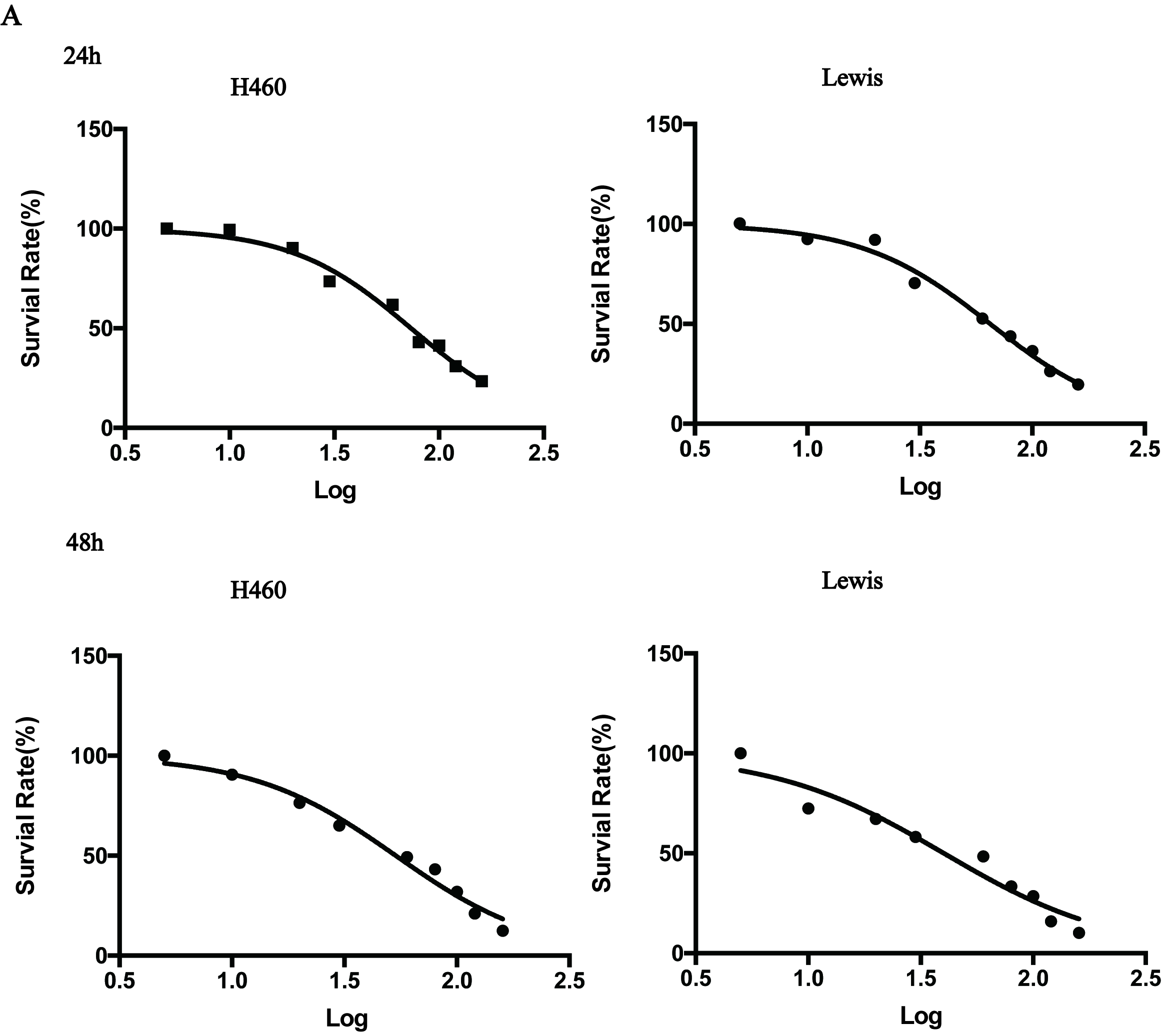

Supplement: Supplementary Figure 2 — Sophoridine inhibited the growth of lung cancer cells in dose-dependent manners. [file Image_2.tif]

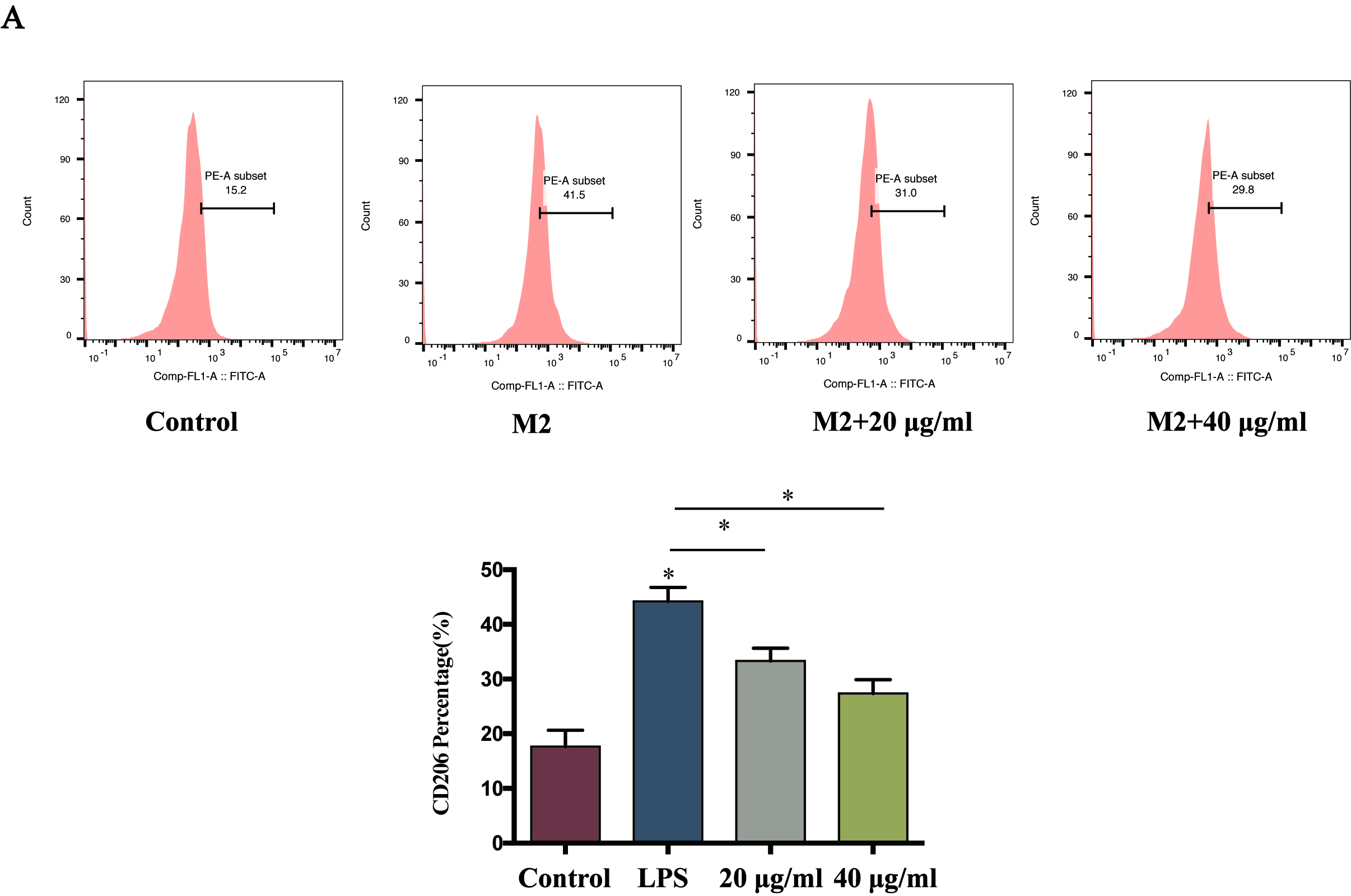

Supplement: Supplementary Figure 3 — Sophoridine suppressed M2 polarisation of macrophages in vitro. [file Image_3.tif]
